# Detecting and attributing the causes of biodiversity change: needs, gaps and solutions

**DOI:** 10.1098/rstb.2022.0181

**Published:** 2023-07-17

**Authors:** Eden Tekwa, Andrew Gonzalez, Damaris Zurell, Mary O'Connor

**Affiliations:** ^1^ Department of Zoology and Biodiversity Research Centre, The University of British Columbia, Vancouver, British Columbia, Canada V6T 1Z4; ^2^ Department of Biology, McGill University, Montreal, Quebec, Canada H3A 1B1; ^3^ Hakai Institute, Heriot Bay, British Columbia, Canada V0P 1H0; ^4^ Institute for Biochemistry and Biology, University of Potsdam, 14469 Potsdam, Germany

**Keywords:** biodiversity, detection, attribution, assessment, global biodiversity framework

## Abstract

This issue addresses the multifaceted problems of understanding biodiversity change to meet emerging international development and conservation goals, national economic accounting and diverse community needs. Recent international agreements highlight the need to establish monitoring and assessment programmes at national and regional levels. We identify an opportunity for the research community to develop the methods for robust detection and attribution of biodiversity change that will contribute to national assessments and guide conservation action. The 16 contributions of this issue address six major aspects of biodiversity assessment: connecting policy to science, establishing observation, improving statistical estimation, detecting change, attributing causes and projecting the future. These studies are led by experts in Indigenous studies, economics, ecology, conservation, statistics, and computer science, with representations from Asia, Africa, South America, North America and Europe. The results place biodiversity science in the context of policy needs and provide an updated roadmap for how to observe biodiversity change in a way that supports conservation action via robust detection and attribution science.

This article is part of the theme issue ‘Detecting and attributing the causes of biodiversity change: needs, gaps and solutions’

## 1. Introduction

In this issue, we address the urgent challenge of assessing biodiversity [1] in an era of accelerating extinction and resource depletion [2,3]. The papers assembled here showcase the rapid development of new technological, statistical and modelling methods needed to observe, detect and attribute the complex array of environmental and anthropogenic causes of biodiversity change. This theme is grounded on issues at the science-policy interface. Important components of this interface include identifying policy pathways, quantifying progress towards conservation goals, and revising estimates of biodiversity change at different spatial and temporal scales. A principled approach to this challenge can also resolve scientific debates about the direction and magnitude of human and environmental impacts on biodiversity [4–6]. Our goal is to provide a roadmap for conducting robust and replicable policy-focused biodiversity assessments.

Today, we know that biodiversity change is implicated in the sustainability of food provision, ecosystem regulation, habitat protection, carbon sequestration, cultural identity and tourism [7]. Ecological theories and empirical studies show that biodiversity is critical for providing ecosystem services [8–10], but that different facets of biodiversity are important for their persistence and resilience in the long term [1,11,12].

Efforts to properly account for the value of nature within our economies (e.g. natural capital accounting and inclusive wealth [13,14]) are challenging because traditional economic theories ignore or incompletely incorporate the dynamics of biodiversity arising from unsustainable exploitation and anthropogenic stress. A consequence of this is that both the science and implementation of biodiversity assessments have received less attention and investment than they deserve in economic and financial activities [13,15]. Without accurate assessments, it has and will continue to be difficult to account for the state and value of biodiversity, attribute change to anthropogenic drivers and conservation efforts, and incentivize a transformation of our economic paradigm that would equip societies confronted with an uncertain future.

Developments linking biodiversity monitoring and assessments are occurring rapidly in response to the need for actionable knowledge. The impetus for this knowledge comes from the need to track and guide progress towards biodiversity goals, notably the United Nations Sustainable Development Goals [16] and the Kunming-Montreal Global Biodiversity Framework [17] at an international level. For example, the International Union for Conservation of Nature red list continually tracks the status of endangered species that leads to legal protection mandates [18]. Fishery science has motivated the creation of sustainability laws including international Exclusive Economic Zones [19], the High Seas Treaty [20] and the Magnuson Stevens Fishery Conservation and Management Act in the United States [21]. In support of these direct policy implementations, other organizations promote standards for the detection and attribution of biodiversity change. The Intergovernmental Science-Policy Platform on Biodiversity and Ecosystem Services provides periodic expert assessments of the state of biodiversity and ecosystem benefits to people [22]. The Group on Earth Observations Biodiversity Observation Network (GEO BON) organizes collaborative international research to generate and share biodiversity data, and provide guidelines for monitoring and the workflows for indicator production [23].

The challenges of effective biodiversity change assessments go beyond economic incentives and investment in monitoring networks. Designing sampling protocols, identifying metrics, correcting estimation bias, quantifying uncertainties, attributing causes, projecting future pathways and designing policies are components that contain major and often underappreciated knowledge gaps. These gaps were contributing factors to the failure to achieve earlier international targets to halt biodiversity loss by 2020 [24]. In addition, incorporating diverse perspectives will be critical in achieving scientific progress and addressing real-world biodiversity problems [25–27]. This issue includes contributions from Asia, Africa, South America, North America and Europe, Indigenous perspectives, early-career scientists (10 papers) and women/non-binary lead authors (seven papers). We hope this journal issue will galvanize the scientific community to more effectively and rapidly assess the changing state of biodiversity, which is critical for achieving national and global biodiversity and sustainable development goals.

## 2. From observation to policy: concept and definition

**Figure 1.**
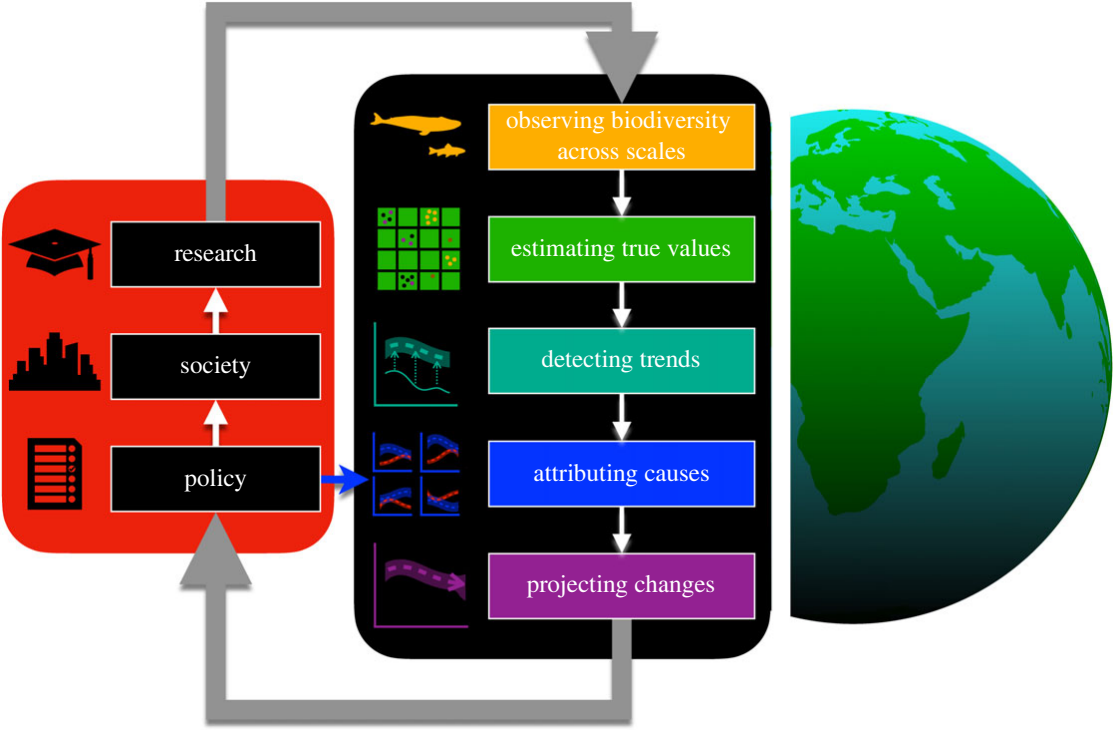
Connections between biodiversity and society. Social actions (left box) lead to demands for assessments and monitoring of the state of biodiversity (right box), which include the detection and attribution workflow required to make robust conclusions. The cyclical flow of information and knowledge between biodiversity science and society is complicated by policies positively or negatively influencing the states of nature and socioeconomic development at different temporal and spatial scales. Through these connections, biodiversity scientists, policymakers and monitoring networks work together to implement rapid assessments, enhance research capacity and respond to urgent knowledge needs. (Online version in colour.)

**Figure 2.**
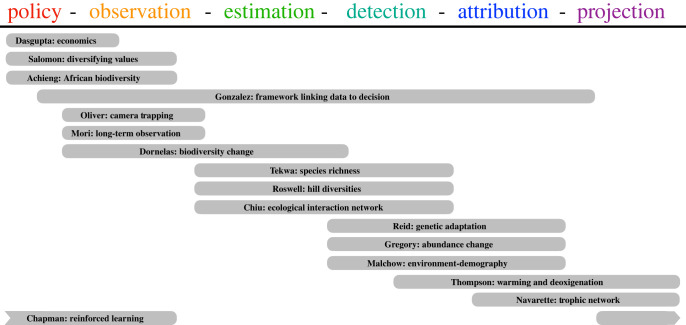
Contributions to the links from biodiversity policy to projection in this issue. (Online version in colour.)

This issue is organized through a conceptual framework linking social and policy needs to the scientific components that underpin robust assessments of biodiversity change (figures 1 and 2).

We identify observation, estimation, detection, attribution and projection as major scientific components of biodiversity assessment [28]. Observation involves measuring the variables that directly or indirectly assess the state of biodiversity in biological communities. Observations may derive from systematic monitoring on the ground, or remotely from space, but increasingly from informal observations made by local communities and citizens [29]. Metrics are derived from theme observation, such as species richness, composition, population abundance, species interaction networks, genetic diversity and ecosystem extent. Estimation is the statistical process of estimating the value of these biodiversity metrics (including estimates of uncertainty) in a place and time given imperfect observations [30]. Detection, by contrast, is the quantification and test of whether a biodiversity metric changes in value across communities over time—for example, a linear or nonlinear trend in the measure of diversity [31]. Our confidence in the estimate of detected change involves propagating uncertainties and constructing expected outcomes from a null hypothesis. Attribution identifies the possible causes of biodiversity change, and tests alternative causal models that include abiotic variables like temperature and precipitation, and anthropogenic variables like nutrient run-off and land use policy [32,33]. Finally, projection uses our knowledge of causal relationships to predict how biodiversity will change given abiotic and anthropogenic stressors. From observation to projection, short-circuiting any component can result in predicting changes in the wrong direction (false positive) or predicting no change when in fact there will be a change (false negative). By focusing on a robust workflow, we hope to reduce errors in inference to better guide policies for conservation and assessments of current and future risk.

We also identify the components of policy, community and research that need better integration. Policy on stewarding and exploiting biological resources is driven by a combination of consumer demand [34], tradition [35], national economic planning [13] and political incentive [36]. All of these motivations can be partly informed by scientific knowledge co-production, ideally from projections of biodiversity change that are unbiased, precise and accurate. Policy influences the goods and harms to society both present and into the future [37], but questions remain on how policy influences consumer choice [34], social norm [38], participation [39] and access [40]. It is also unclear how society and public opinion will drive the questions, funding, technology and infrastructure shaping biodiversity research capacity to drive policy in turn [41]. These social topics should help scientists and policymakers chart the paths towards continually updated biodiversity stewardship.

The natural and social sciences covering these biodiversity issues span almost all disciplines, so it would be impossible for a single journal issue to cover all important aspects. As well, the complexity of socioecological dynamics prevents a complete synthesis from being feasible at the moment (acyclical information flows in figure 1). Given these constraints, we sought to feature a set of perspective, methodological, empirical and theoretical papers representing state-of-the-art and policy-relevant tools that enable tracking progress towards biodiversity and sustainability goals. In particular, we focus on the scientific developments that enable assessment and communication, and in addition highlight how these scientific tools can inform and be motivated by the greater societal context of policy. The 16 contributions highlight the diverse voices, geographic issues, methodological challenges and study systems that shape the field today.

## 3. Contributions summary

We ordered the contributions to this issue according to the major information flows through the compartments of society and biodiversity science (figure 1). The contributions are divided into four major themes: *policy motivations and scientific frameworks, observation networks, statistical estimation and detection,* and *attribution and projection*.

### (a) Policy motivations and scientific frameworks

Three perspectives in this issue, Dasgupta & Levin [42], Salomon *et al.* [43], and Achieng *et al*. [44] remind us that humanity is inseparably embedded in nature. These papers motivate the need to invest in biodiversity science and incorporate diverse knowledge types and values throughout society. To address these social challenges, Gonzalez *et al*. [28] provide a scientific framework for detecting and attributing biodiversity change in the context of anthropogenic and environmental variations.

Dasgupta & Levin [42] argue that contemporary economic thinking which guides our major global policies is based on an unsound understanding of the finiteness of our planet's natural resources. This perspective ignores the growing exposure of our economy to the risks of unsustainable biodiversity loss and loss of natural capital and the benefits society accrued from it. They argue that we must shift the emphasis from gross domestic profit (GDP) to measures of inclusive wealth that account for natural assets, not just capital assets. National statistical offices should track inclusive wealth and its distribution, not GDP and its distribution. Armed with the concept of inclusive wealth, they identify available policy instruments that can greatly improve the management of global public goods such as the oceans and tropical rainforests. They also point to biodiversity impacts via global trade liberalization where ecosystems in developing nations are impacted by the products extracted and harvested by developing countries, leading to a transfer of inclusive wealth to rich importing countries. Humanity's embeddedness in nature implies a whole-of-society transformation is needed, one that recognizes, monitors and mitigates the complex causal pathways that are impacting our biosphere from the smallest to largest scales.

Salomon *et al*. [43], a group that includes 10 Indigenous community leaders, bring our attention to how colonization overtook existing perspectives on nature that included humans, and how biodiversity science actually played a role in colonization. Their perspective draws on governance principles from 17 Indigenous nations from the northwest coast of North America to highlight additional disconnects between conventional scientific perspectives on biodiversity change and the integrated view of people and nature that many Indigenous cultures lived with for thousands of years prior to colonization. They offer clear suggestions for moving forwards with biodiversity change policies by including multiple perspectives in biodiversity assessment activities and considering multiple beneficiaries of biodiversity conservation.

Continental variations in biodiversity and assessment capacity are illustrated by Achieng *et al*. [44]. The authors highlight that biodiversity hotspots in Africa today may be experiencing relatively severe impacts from social developments and global trade liberalization compared to other continents, and therefore should be assessment priorities. Yet most of Africa currently has a poor capacity to systematically collect data because of economic, political and academic constraints, problems that may or may not be solved by international investments.

In a synthesis paper, Gonzalez *et al*. [28] show that biodiversity change is ever-present and can involve multiple interacting facets of biodiversity (e.g. genetic, species and ecosystem diversity). Biodiversity fluctuates over time in ecosystems that are unimpacted by human drivers. To understand biodiversity change in a rigorous way that allows for quantitative comparison and discussion of causes attribute to human drivers, Gonzalez *et al*. [28] argue that formal standards for detecting changes and attributing changes to causal drivers are essential in the context of assessments and policy discussions. A similar framework played a pivotal role in climate change science, moving the discussion from whether climate change was caused by human activities or not to a discussion of how to mitigate or reverse it. Detection and attribution of biodiversity change are challenging because the different facets of nature, and what they mean to people, can be measured in different ways with varying precision. The paper lays out in detail five key steps in the scientific framework for the detection and attribution of biodiversity change that allow strong inferences to be made about the drivers responsible for the patterns of change observed over space and through time (figure 1). The authors close with a call to the research community to come together to work on the monitoring and modelling standards required to scale up our capacity to do detection and attribution research worldwide.

### (b) Observation networks

Biodiversity observations are vital for assessing how policies and environmental and anthropogenic pressures impact biodiversity. Yet, most biodiversity data are biased geographically, taxonomically or thematically. Oliver *et al*. [45] explore the potential of camera trap surveys to increase knowledge of the status and trends of species in terms of distribution and abundance. Focusing on mammals and birds, the authors show that continuous-time camera trap surveys expanded taxonomic coverage and spatial coverage compared to publicly available data from the Global Biodiversity Information Facility. Camera traps are only one example of novel technologies for biodiversity observations. Imaging and acoustic sensors are developing rapidly and so is computer vision for automated image recognition. As Oliver *et al*. [45] clearly demonstrate, these new sensors open exciting opportunities for filling knowledge gaps in biodiversity data.

Mori *et al.* [46] advocate long-term, fine-resolution ecosystem observations to understand the complex feedbacks that natural and anthropogenic drivers can induce in food webs and ecosystem functioning. Drawing on examples of terrestrial and marine monitoring in Japan, they discuss important barriers and challenges to ecosystem monitoring, for example, related to funding and coordination, and the joint monitoring of biotic and abiotic factors. Importantly, Mori *et al.* [46] suggest that inclusive and equitable collaborations should be established to provide training in cutting-edge technologies such as laser imaging detection and ranging (LIDAR) and environmental DNA (eDNA) for developing countries. This would be a cornerstone for a standardized and equitable global observation network.

Biodiversity assessments hinge on identifying metrics most indicative of nature's states and changes under climate and anthropogenic stressors. While essential biodiversity metrics have been identified, it is still an open question as to what are the most important metrics and how they impact ecosystems and societies. Dornelas *et al*. [47] provide a perspective on the current evidence of changes in commonly tracked biodiversity metrics. They suggest that evidence for temporal change in species richness and spatial beta-diversity (measuring differences between local communities) are ambiguous at both local and regional scales; by contrast, there are stronger signals of changes in compositional turnover and abundance. Although the authors did not focus on theories explaining how these aspects of biodiversity should respond to ongoing environmental and anthropogenic stressors, the empirical evidence suggests species richness may be stable and resilient regardless of its role in determining ecosystem dynamics. By contrast, other biodiversity metrics may more strongly respond to or determine ecosystem dynamics under stress. From a policy perspective, biodiversity metrics are important insofar as they indicate nature's potential to maintain ecosystem services. The open questions remain which metrics are the best indicators of different aspects of ecosystem services, and how these aspects are changing in the Anthropocene.

### (c) Statistical estimation and detection

The lack of evidence for changes in some biodiversity metrics may mean they really are not associated with changes in ecosystem functions, but observational and statistical deficiencies may also lead to a lack of power to detect biodiversity changes even if they are occurring. In line with open empirical questions about biodiversity indicators, Roswell *et al*. [48] explore whether species richness or other biodiversity metrics (Hill's diversity spectrum) that weigh richness and evenness differently (including the Simpson and Shannon indices) show stronger relationships with ecosystem functioning in case studies. Although Hill's diversities that weigh evenness more than species richness should suffer less statistical biases from limited data and imperfect observations, the authors find that richness is still the strongest indicator of ecosystem services. These results show that improving our ability to infer true richness and detect richness changes remain primary research objectives besides tracking other biodiversity metrics.

Our issue identifies two essential biodiversity metrics with strong theoretical roles that are sensitive to observational biases, with current correction methods remaining suboptimal to accurately assess biodiversity states and changes using data from most monitoring programmes today. These metrics are species richness [49] and ecological interaction network diversities [50], each of which is expected to respond to and determine ecosystem functioning under environmental and anthropogenic stresses. Even if richness itself is expected to be stable over time as suggested by current evidence [47], estimating the number of missing species in surveys is critical to assess how much information and additional sampling effort are required to accurately measure other biodiversity metrics that hinge on identifying all species in a community. Tekwa *et al*. [49] present a new method that uses rarity and information about spatial heterogeneity already contained in common biodiversity surveys. The new estimator provides a superior balance of bias, precision and accuracy to simultaneously estimate true richness and detect differences between communities or time points relative to other methods. However, the authors show that when the actual richness change is small, common surveys have low statistical power to detect change using any current correction methods. These results illustrate the often underappreciated need to invest in biostatistical research.

Identifying actual species interactions is impossible without having observed all extant species in a community, but Chiu *et al*. [50] show it is possible to partially recover missing summary information such as the number of unobserved species interactions to infer ecological network diversities. While theories predict how network diversities in communities should respond to stress, they remain untested with empirical data. The work by Chiu *et al*. [50] represents an important step in reaching the goals of testing hypotheses and monitoring a recently identified aspect of biodiversity.

### (d) Attribution and projection

Reid *et al*. [51] begin with the observation that many wild populations may only be able to persist through rapid evolutionary adaptation to novel conditions created by humans. In fisheries, evolution may occur in response to harvesting. Adaptation may occur via shifts in the frequency of a few genes of large effects or via polygenic adaptation through shifts in many genes of small effects. Detecting the adaptive evolutionary processes responsible for the persistence of threatened populations is key to long-term detection and attribution research. Reid *et al*. [51] assess which of these alternatives may have occurred in Atlantic cod (*Gadus morhua*) using a spatially replicated dataset of temporal genomic data combined with model simulations. They find evidence for harvesting-induced evolution via polygenic adaptation and trait selection sustained over several decades. However, attributing harvesting with high confidence for the patterns of polygenic trait variation is not easy and will require a combination of spatially replicated population genomic time series in contrasting selective environments combined with models to provide expectations for patterns of genetic covariance in allele frequency over long time periods. This is an exciting challenge for future detection and attribution research on genetic diversity, and its links to population persistence, that extends far beyond harvesting in marine populations.

Gregory *et al*. [52] report an analysis of trends in the abundance of native breeding birds in the UK and Europe. This reflects an analysis up to the detection step in the detection and attribution framework (figure 1). They deploy a Bayesian hierarchical time series model to estimate trends among species. They find significant changes in the bird assemblages. Abundance trends across species are positively correlated with species' body mass and with trends in climate suitability, which vary with species’ abundance, migration strategy and niche associations linked to diet. Their work highlights how changes in biodiversity cannot be captured easily by a single number. However, their analysis stops short of formal trend attribution. A key message in the context of detection and attribution is that care is required when measuring and interpreting biodiversity change given that different metrics can provide very different insights into the changing state of biodiversity at different jurisdictional levels and spatial scales.

Malchow *et al*. [53] analyse trends in the distribution and abundance of different Swiss breeding birds using spatially explicit, mechanistic models. Models are calibrated within a Bayesian statistical framework and jointly considered the effect of dispersal and demographic processes on observed range and population dynamics. The key novelty in their approach is the estimation of demography–climate relationships for different demographic rates that allows the identification of climate signals in recent population response. For the specific case study, results suggest that fecundity and juvenile survival depended more strongly on climate than adult survival, and that recent population decreases of mountain species had actually been buffered by climate change, although more adverse effects of climate change can be expected in the future. The authors demonstrate the feasibility of fitting complex, mechanistic simulation models to monitoring data. Placing such dynamic and mechanistic models in a solid statistical framework allows for attributing observed biodiversity trends to different abiotic and biotic drivers and assessing transient dynamics and lagged responses.

Thompson *et al*. [54] use fishery assessment and environmental data from trawl surveys to attribute factors that determine groundfish habitats on the west coast of North America. Subsequently, these attributions, including the effects of temperature and oxygen, are used to project future groundfish biodiversity change across space and water depth under the changing conditions predicted by ocean models. This work exemplifies a methodology that links detected biodiversity differences with attributing potential causes and projecting future changes that serve as testable hypotheses and inform policy.

Navarette *et al*. [55] illustrate how monitoring complex communities might be enhanced through dynamic, quantitative modelling that emphasizes species interactions as the important ‘units’, or foci, for monitoring. Working with an intertidal kelp harvesting system in Chile, their approach considers how species interactions respond to ecological change and to management actions. Their approach goes beyond the use of indicator species, which they argue can miss the critical dynamics that link ecological function with desired outcomes such as productivity to fishers. They show, through the use of a dynamical model, how a set of potential causes of biodiversity change (harvesting, compliance with regulations) can manifest in different outcomes for fishers and managers. Such approaches are an essential feature of the causal analysis framework in the detection and attribution framework introduced by Gonzalez *et al.* [28].

The interactions between humans, nature, and environmental change are complex and capable of producing unexpected dynamics. Therefore, process-based models of how to manage biodiversity change may be theoretically intractable and empirically unidentifiable in many social–ecological systems. For example, the socioecological coupling of fish population dynamics, economics and management produces the alternative stable states of conservation or overexploitation, which are indistinguishable if we only measure derived ecosystem services (e.g. revenue from consumption) without independent stock assessments, which may not be available. Chapman *et al*. [56] suggest one way forward is harnessing artificial intelligence through reinforcement learning from multiple observations and models to guide management action when we cannot infer underlying social–ecological processes. Still, the reinforcement learning process relies on a comprehensive portfolio of empirically motivated, process-based scenario models. This highlights the importance of the statistical and theoretical approaches taken by other contributions in this issue, even when we face the seemingly intractable complexity of biodiversity that requires a diversity of novel thinking.

## 4. Looking forward

Although our issue has covered some major themes in quantifying and understanding biodiversity change, some emerging issues were not addressed. These missing links include incorporating genetic diversity [11], spatial planning and protected areas [57], protecting and restoring connectivity [58], harnessing mechanistic scenario-based models and assessing ecosystem impacts [59], considering the ethics of sampling and monitoring for local cultures and organisms [60], and developing fundamental theories relating different sources and measures of biodiversity-function and -stability relationships [10,61]. Systematic workflows for conducting policy-oriented biodiversity assessments have yet to incorporate information derived from monitoring [1], in contrast to climate research where standard protocols for research and data sharing have been largely established by scientific census [62,63]. Protocols are also needed to standardize novel sensor-based community data including eDNA [64], camera trapping and remote sensing, and to facilitate effective data integration with other data sources, similar to what has been established in genomic research [65].

Causal analysis in biodiversity science is still in its infancy, partly because statistical and mechanistic modelling tools are underdeveloped given the complexity of ecological systems [66–68], or relevant methods have not been widely adopted from other fields [69–71]. Current biodiversity modelling frameworks are strongly biased towards correlative models and the population or species level, and are missing key biological processes [72,73]. This correlative bias limits our ability to understand drivers of, and coupling between, multiple essential biodiversity variables [23] across ecological levels. Correlative models without understanding biological processes will limit or inflate our confidence in projections across environmental change scenarios. In addition, the selection of potential causes of biodiversity change is invariably subjective and incomplete. Thus, acknowledging motivations and improving inclusiveness in biodiversity science not only address more accurate natural capital accounting and forecasting, but also help discover solutions that can be implemented among diverse social communities [26,41,74,75].

We hope this issue provides guidelines for linking biodiversity observations, monitoring and inferences about the rates and reasons for biodiversity change. A robust detection and attribution framework will inform the implementation of policies designed to protect, manage and sustain biodiversity and ecosystem benefits at the heart of the Kunming-Montreal Global Biodiversity Framework [17] and the UN Sustainable Development Goals [16].

## Data Availability

This article has no additional data.
